# Challenges in the Management of Giant Carcinoma Ex-Pleiomorphic Adenoma of the Parotid Gland in a Single Tertiary Center

**DOI:** 10.3390/medicina61010037

**Published:** 2024-12-29

**Authors:** Miruna Bratiloveanu, Mihai Dumitru, Andreea Nicoleta Marinescu, Crenguta Serboiu, Oana Maria Patrascu, Adrian Costache, Daniela Vrinceanu

**Affiliations:** 1 Pathology Department, Carol Davila University of Medicine and Pharmacy, 050474 Bucharest, Romania; mirunabratiloveanu@gmail.com (M.B.); oanamaria.patrascu@gmail.com (O.M.P.); adriancostacheeco@yahoo.com (A.C.); 2 ENT Department, Carol Davila University of Medicine and Pharmacy, 050751 Bucharest, Romania; vrinceanudana@yahoo.com; 3 Imaging Department, Carol Davila University of Medicine and Pharmacy, 020021 Bucharest, Romania; andreea_marinescu2003@yahoo.com; 4 Molecular Biology and Histology Department, Carol Davila University of Medicine and Pharmacy, 050474 Bucharest, Romania; crengutas@yahoo.com

**Keywords:** parotid, gland, pleiomorphic, adenoma, carcinoma

## Abstract

*Background and Objectives*: Carcinoma ex-pleiomorphic adenoma (CXPA) is a carcinoma derived from a primary or recurrent pleiomorphic adenoma. Microscopically, non-invasive CXPA (intracapsular and carcinoma in situ), minimally invasive CXPA (extracapsular invasion less than 1.5 mm), and invasive CXPA (extracapsular invasion more than 1.5 mm) are described. *Materials and Methods:* We performed a retrospective clinical study over the period of 2009–2023 in patients admitted to the ENT Department of the Bucharest University Emergency Hospital. *Results:* In the studied group, there was a net male predominance of 2.5:1. The tumor evolution until presentation was 6.64 years on average, with values between 2 and 20 years. The reasons for presenting to our department included a sudden increase in size in eleven cases (78.57%), pain in nine cases (64.29%), peripheral facial paralysis in eight cases (57.14%), skin invasion/ulceration in five cases (35.71%), and massive tumor hemorrhage in one case (7.14%). There were histopathological results on paraffin of myoepithelial CXPA in four cases (28.57%), of high-grade CXPA (salivary duct, secretory) in eight cases (57.14%), and of squamous CXPA in two cases (14.29%). The patients with unfavorable evolution showed the following characteristics: a tumor diameter over 11 cm (four cases), integument invasion (four cases), perivascular invasion at HP exam (six cases), perineural invasion at HP exam (six cases), and invasion of the ganglion (three N3b cases and two N1 cases). *Conclusions:* CXPA is a neoplasia that, when associated with large tumor volumes or peripheral facial paralysis in particular, is a challenge for both the doctor and patient.

## 1. Introduction

Carcinoma ex-pleiomorphic adenoma (CXPA) is a carcinoma derived from a primary or recurrent pleiomorphic adenoma. It represents the most common variant of mixed malignant tumors, the other types being carcinosarcoma and pleiomorphic adenoma metastasis. Over 75% of these tumors occur in the parotid gland, but they can occur in all other types of salivary gland, major or minor. CXPA accounts for 3.6% of all salivary gland tumors, 6.2% of all mixed salivary gland tumors, and 11.6% of all malignant salivary gland tumors [1]. The prevalence rate is 5.6 cases/100,000 malignant tumors and 0.17 cases/1,000,000 people/year. It occurs more frequently in those aged between 60 and 80 years and more frequently in women. Geographical differences in the distribution of CXPA have also been noted in the literature [2]. In the UK, CXPA accounts for 25% of primary parotid malignancies. In the USA, CXPA has a prevalence of 12% among primary parotid malignancies. In Denmark, malignant transformation occurs in 3.2% of recurrences. It is, therefore, a rare type of tumor that represents a diagnostic and therapeutic challenge [3].

Clinically, a long-evolving hidden parotid gland tumor that suddenly increases in size can lead to pain, peripheral facial paralysis, and lymph node metastases, as well as skin infiltration and ulceration. One in four patients with such a tumor had a previously treated pleiomorphic adenoma [4]. Peripheral facial paralysis occurs in up to 30% of cases, and lymph node metastases in up to a quarter of cases. In advanced cases, skin ulceration, palpable adenopathy, dysphagia, and mandibular invasion are present. The long duration of evolution is characteristic of this type of tumor: an interval from 1 month to 52 years is described in the literature, with the average duration of evolution currently estimated to be 9 years [5].

Advanced imaging is represented by CT scans with IV contrast, which can highlight mandibular or temporo-zygomatic bone lesions. The gold standard is cervical MRI, which is the most sensitive in detecting malignancies and enables a clearer delineation of tissue planes. In this regard, MRI with diffusion-weighted sequence and ADC is very useful for highlighting areas of malignancy [6].

Ultrasound-guided fine needle aspiration (FNA) cytology has a sensitivity and accuracy of 50–64% and 40%, respectively, so it is used less often in the diagnosis of CXPA, especially if the clinical criteria of malignancy and large areas of tumor necrosis are met. In addition, FNA carries a high risk of false negative results. A histopathological and immunohistochemical examination of the surgical specimen enables a more certain diagnosis [7].

Macroscopically, a gray-blue, transparent, or yellowish-white pleiomorphic adenoma component can be observed in a sample of resected CXPA, alongside the malignant, infiltrative component, with necrosis and hemorrhages [8]. Microscopically, the tumor is composed of a mixture of pleiomorphic adenoma and carcinoma. In the territory of pleiomorphic adenoma, foci of malignancy are visualized, with nuclear pleomorphism, frequent or atypical mitoses, hemorrhages, and necrosis. The malignant component is most commonly adenocarcinoma, but it can also be adenoid cystic carcinoma, mucoepidermoid carcinoma, salivary duct carcinoma, or myoepithelial carcinoma [9]. In lymph node metastases, only the carcinomatous component is identified. Microscopically, non-invasive CXPA (intracapsular and carcinoma in situ), minimally invasive CXPA (extracapsular invasion less than 1.5 mm), and invasive CXPA (extracapsular invasion more than 1.5 mm) are described [10].

A differential diagnosis of CXPA is made by excluding other benign or malignant salivary gland tumors. These differ from true mixed tumors and are represented by carcinosarcomas, which have only purely epithelial elements or epithelial and mesenchymal elements. CXPA must also differentiated from metastatic mixed tumors that do not have a carcinomatous component [11].

The surgical management of malignant tumors of the parotid gland involves total parotidectomy (levels I–IV) associated with elective neck dissection in CN0 + risk factors for occult metastases or with therapeutic neck dissection in CN+, advanced T stage, and high-grade tumors with facial paralysis. Adjuvant radiotherapy is indicated in high-grade forms with inadequate resection, perineural invasion, and lymph node metastases [12].

Prognostic factors in CXPA are represented by the tumor size and grading (a high grade indicating a poorer prognosis), level of invasion, nodal and distant metastases, long evolution, and incomplete resection. Locoregional recurrence is a major prognostic factor and is correlated with a median survival of less than 1 year. The 5-year survival in CXPA patients is, according to the literature, between 25 and 65% [13].

The aim of the present study was to communicate our experience in the management of giant CXPA of the parotid gland in a tertiary head and neck center, motivated by the rarity of this type of tumor and its particular clinical evolution compared to other malignant tumors of the parotid gland.

## 2. Materials and Methods

We performed a retrospective clinical study on patients admitted to the ENT Department of the Bucharest University Emergency Hospital between 2009 and 2023. We included patients with complete medical records and microscopic diagnosis of CXPA. We identified 14 patients with a histopathological diagnosis of CXPA of the parotid gland and giant tumor masses on admission. We excluded patients with other parotid gland tumors such as pleiomorphic adenoma with atypia, carcinosarcoma, and other types of parotid gland carcinomas, as well as other parotid gland neoplasms (carcinoma metastases and lymphomas). We studied the distribution of these patients according to sex, age, duration of evolution until presentation, reasons for presenting to our department, type of operation performed, extemporaneous examination (frozen sections), paraffin histopathological examination, and postoperative TNM staging, as well as according to evolution.

## 3. Results

**Figure 1:**
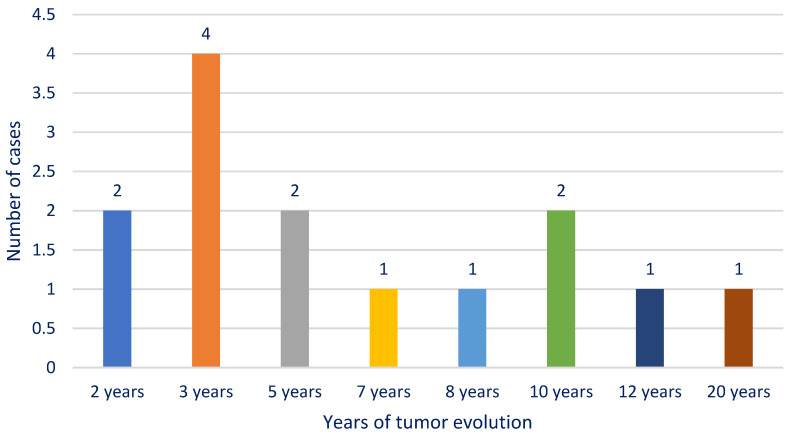
Chart depicting the distribution of cases according to the duration of evolution.

In the study group, we had an M/F ratio of 10/4, with a net male predominance of 2.5:1 (78% male, 22% female). The cases were distributed as follows: four cases in the 51–60-years age group (28.57%); three cases in the 61–70-years age group (21.42%); and seven cases in the 71–80-years age group (50%). The average age was 67.5 years, with a minimum of 53 years and a maximum of 79 years. The highest frequency corresponded to the patients aged 77 years, and the median age was 70. In our department, we observed an average duration of tumor evolution until presentation of 6.64 years, with values between a minimum of 2 years and a maximum of 20 years (Figure 1). The highest frequency in the mode corresponded to an evolution of 3 years (four cases), and the median was 5 years. We also noted a history of operated pleiomorphic adenoma in six cases (42.86%).

**Figure 2:**
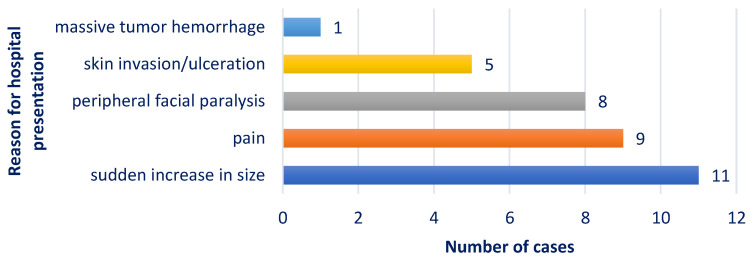
Chart depicting the distribution of cases according to the reason for presentation to ENT Department.

Figure 2 presents the distribution of the study group according to the main symptom leading to the patient’s presentation at the hospital.

The surgery performed in these patients consisted of a total parotidectomy in 10 cases (71.43%); total enlarged parotidectomy in 4 cases (28.57%); Functional Neck Dissection (FND) in 7 cases (50%); Radical Neck Dissection (RND) in 7 cases (50%); and facial nerve resection in 3 cases (21.43%).

**Figure 3:**
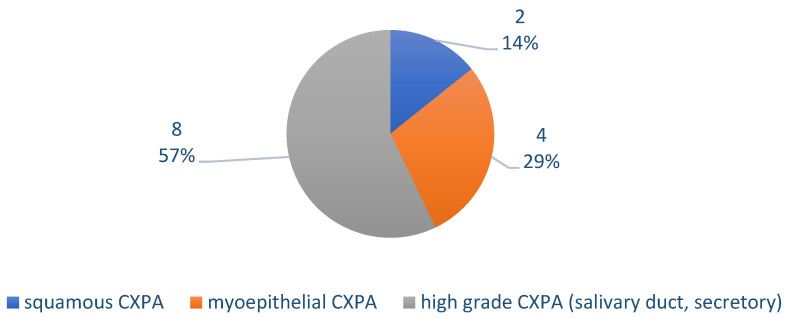
Chart depicting the distribution of cases according to paraffin histopathological examination (absolute and relative values).

**Figure 4:**
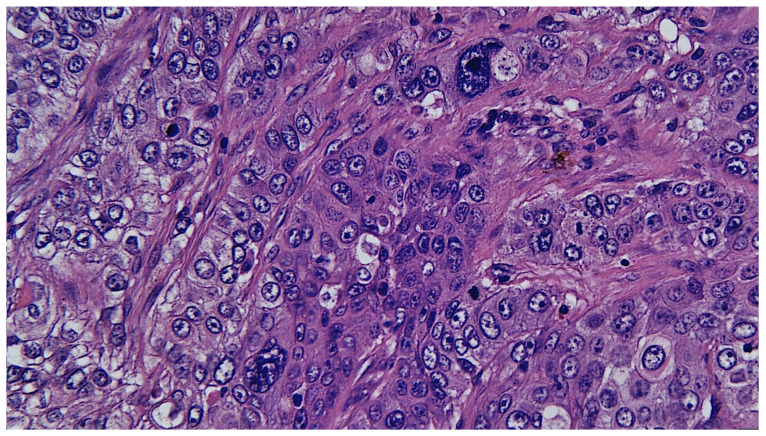
Cellular detail of a salivary duct carcinoma arising on a pleiomorphic adenoma (pleiomorphic ex-adenoma carcinoma combined with salivary duct tumor). Significant pleomorphism is seen with irregular, large nuclei with anisokarya and multiple, evident nucleoli. In places, multinucleated cellular monstrosities are observed. Cytoplasm shows marked eosinophilia; HE stain, 40×.

**Figure 5:**
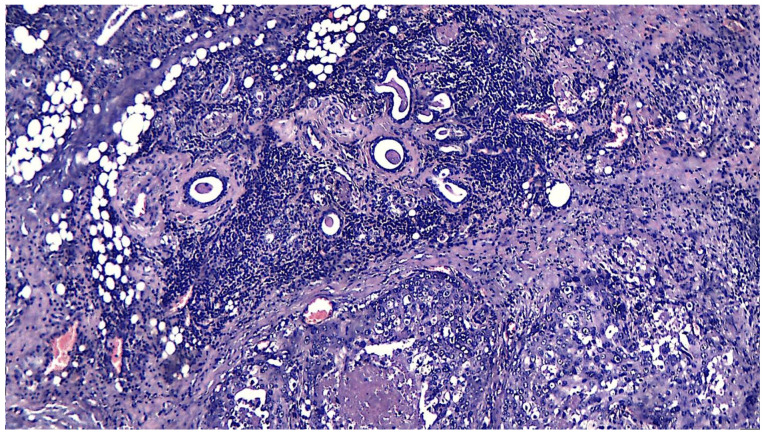
Salivary duct carcinoma appearing in a pleiomorphic adenoma (APP patient). Poorly differentiated tumor infiltration is observed, with the formation of glandular and cribriform structures in a fibro-hyaline stroma with peritumoral lymphoplasmacytic inflammatory infiltrates, with comedonecrosis; HE staining, 20×.

**Figure 6:**
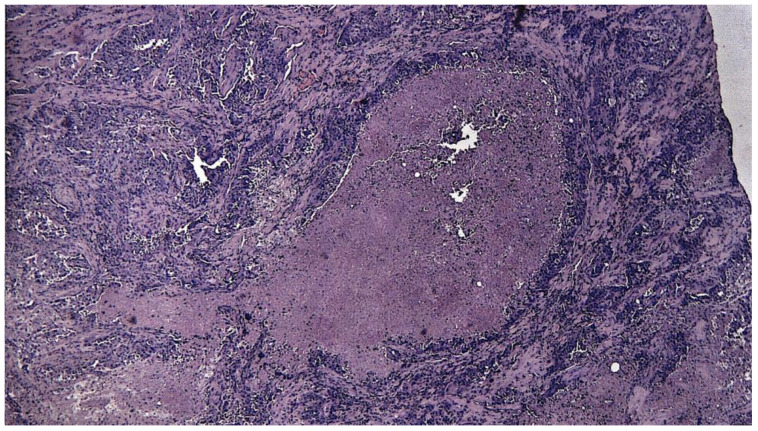
Salivary duct carcinoma arising on a pleiomorphic adenoma (APP patient). Poorly differentiated tumor infiltration is observed, with the formation of glandular and cribriform structures in a fibro-hyaline stroma with significant comedonecrosis; HE staining, 4×.

Figure 3 presents the distribution of cases according to the paraffin histopathological examination. Microscopically, the CXPA was invasive in 13 cases (92.86%) and 1 case appeared to have minimally invasive CXPA (7.14%) (Figure 4). The histopathological elements observed were as follows: capsule breach in all 14 cases (100%), lymphovascular invasion in 11 cases (78.57%) (Figure 5), perineural invasion in 9 cases (64.29%) (Figure 6), and lymph node invasion in 6 cases (42.86%) (pn+ = 6). Of these, there were three N1 cases and three N3b cases (with ENE+ extranodal extension).

**Figure 7:**
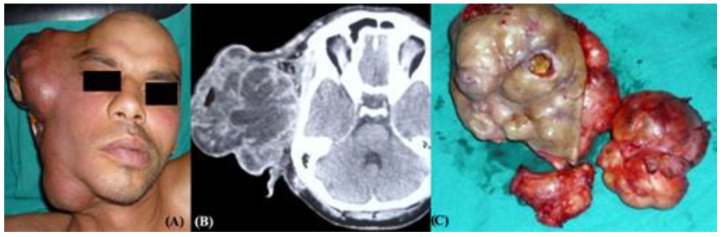
T4-stage CXPA. (**A**) Clinical image of giant right CXPA; (**B**) CT scan with contrast media; and (**C**) complete surgical resection piece.

The TNM stages according to T were as follows: pT2 = five cases (35.71%), pT3 = five cases (35.71%), and pT4 = four cases (28.57%). The staging according to N was as follows: pN0 = eight cases (57.14%) and pN+ = six cases (42.86%). The clinical staging after M revealed cM0 in all 14 cases (100%) (Figure 7).

Facial nerve resection was performed in only three cases in which perineural tumor invasion was macroscopically evident. In the other five cases, we resected only fillets from the cervico-facial trunk, but we preserved fillets from the temporo-zygomatic trunk so that eyelid occlusion was possible. All of these patients also received adjuvant therapy. 

According to the evolution at 5 years, there was recurrence in eight cases (57.14%) and locoregional recurrence in six cases (42.86%). Among these six cases, four cases were associated with a tumor size T over 11 cm and two cases had a high-grade histology. Distant metastases accompanied locoregional recurrence in all six cases.

The patients with locoregional recurrence and distant metastases showed the following characteristics: tumor sizes over 11 cm (four cases), skin invasion (four cases), perivascular invasion (six cases), perineural invasion (six cases), and lymph node invasion (six cases, of which three cases were N3b ENE+). The presence of minimally invasive carcinoma was associated with 5-year disease-free survival.

## 4. Discussions

Pleiomorphic ex-adenoma carcinoma (CXPA) is a rare pathology that is mainly observed in the parotid gland, but which can basically affect any salivary gland, including the minor salivary glands, although this occurs rarely [14].

In ex-adenoma pleiomorphic carcinoma in combination with salivary duct carcinoma, pleiomorphic adenoma nodules can be observed, with a benign appearance, intertwined with tumor structures with a solid architecture. This is accompanied by the formation of glandulo-cribriform structures, sometimes with ductal structures similar to those of ductal carcinoma in situ of the breast or those arranged in trabeculae and cords, composed of cells with significant pleomorphism, eosinophilic cytoplasm, irregular and obvious nucleoli, and frequent atypical mitoses and anisokarya. The described tumor structures have clear infiltrative characteristics, are surrounded by desmoplastic stroma and may present areas of comedonecrosis [15]. Salivary duct carcinoma represents a highly malignant tumor with a reserved prognosis, frequently presenting at examination extensive lymphovascular or perineural invasion. Histopathologically and immunohistochemically, it resembles invasive ductal carcinoma of the breast and shows positive immunohistochemical expression for Her2neu as well as androgen receptors [16].

Another relatively frequent tumor component in pleiomorphic ex-adenoma carcinoma is represented by myoepithelial carcinoma, a malignant tumor with its origin in the myoepithelial cells of the salivary glands, being considered a tumor of low malignancy. Histopathologically, it is characterized by cellular structures arranged in trabeculae or cords, made up of cells with varied morphology, which may have a plasmacytoid, epithelioid, or fusiform appearance, arranged in a hyaline or myxoid stroma, frequently with a desmoplastic reaction. Invasiveness is evidenced by nodular or multinodular tumor growth with areas of central necrosis [17].

The etiology and pathophysiology of pleiomorphic ex-adenoma carcinoma are not yet fully elucidated. Changes such as the loss of PLAG 1 expression or non-specific Her2neu amplification were highlighted [18].

In terms of molecular pathology mechanisms, CXPA development follows a multistep carcinogenesis model: the progressive loss of heterozygosity on the chromosome arms 8q, 12q, and 17p. There are specific genes in these regions associated with particular stages of CXPA progression. Genes regulating tumor suppression and cell cycle control (p53, cyclin D1, p16, and p21), growth factors (FGF, EGFR, and HER2), and intercellular adhesion (E-cadherin, N-CAM, and beta-catenin) are also involved [19].

According to our statistical analysis, the most numerous tumor types were CXPA with malignant salivary duct tumor in eight cases (57.14%), followed by those with myoepithelial malignant in four cases (28.57%) and squamous carcinoma in two cases (14.29%). Only one case was minimally invasive, while the other 13 cases (92.86%) were invasive. All of the cases were therefore associated with capsule rupture, while lympho-vascular invasion was recorded in 11 cases (78.57%), and perineural invasion in 9 cases (64.29%). Nodal invasion was recorded in six cases (42.86%). Notably, half of the cases with nodal invasion exhibited a N3bENE+ capsular break. Regarding the concordance of the frozen sections, in eight cases (57.14%), the result was CXPA, and, in five cases, it was carcinoma. In only one case, the examination of the frozen sections indicated pleiomorphic adenoma with squamous metaplasia.

Compared to data from the literature indicating a predominance of this tumor type in women, our group indicated a ratio of 2.5:1. Regarding the predominant age group, our data overlap with those in the literature, with seven cases being in the 71–80 age group (50%), and three cases in the 61–70 age group (21.42%), and, so, a total of 10 cases (71.42%) between 60 and 80 years of age. The management plan for CXPA must also be determined according to the patient’s age and the existence of comorbidities. It must also be taken into account that this particular type of neoplasia appears in pleiomorphic adenomas with a long evolution, which sometimes reach gigantic dimensions, and in which malignancy is a stage in its evolution. We found that, according to the clinical history of these patients, a pleiomorphic adenoma was operated on in six cases (42.86%) and the average duration of evolution was 6.64 years.

The most frequent reasons for presenting to the hospital included a sudden increase in size in 11 cases (78.57%), pain in 9 cases (64.29%), and facial paralysis in 8 cases (57.14%), all being signs associated with malignancy. Skin invasion, seen in five cases, and massive tumor hemorrhage, seen in one case, are clinical features associated with malignant parotid gland tumors.

Surgical management in CXPA involves ablation and neck dissection, associated or not with reconstruction. CXPA-type tumor ablation consists of parotidectomy, most commonly total parotidectomy, consisting of deep and superficial lobe ablation with preservation of the facial nerve. Radical parotidectomy involves the resection of the tumoral parotid gland en bloc with the facial nerve, being the therapeutic option in the case of clinical facial paralysis. Superficial parotidectomy (ablation of levels I and II) has extremely limited indications in intracapsular or minimally invasive CXPA [20]. Concomitant neck dissection can be elective, functional, or radical; in the latter case, the ideal variant is in monobloc with the tumor. Reconstruction refers to facial reanimation procedures and soft tissue reconstruction. Facial resuscitation procedures are an essential therapeutic modality for improving the quality of life of CXPA patients with preoperative or iatrogenic facial paralysis. These are not indicated if the peripheral facial palsy is older than 6 months. Soft tissue reconstruction can be performed with a radial free flap, sternocleidomastoid flap, or rotated cervical flap, depending on the substance defect resulting from the ablative time and according to the surgeon’s experience [21].

Usually, these giant tumors with CXPA present very rare histology subtypes such as giant sarcomatoid carcinoma ex-pleiomorphic adenoma of the parotid gland. These giant tumors with a long evolution and can reach diameters of 15 cm, eliciting a major impact on the patient’s quality of life [22]. The patients with unfavorable evolution showed the following characteristics: a tumor diameter over 11 cm (four cases), integument invasion (four cases), perivascular invasion at HP exam (six cases), perineural invasion at HP exam (six cases), and invasion of the ganglion (three N3b cases and two N1 cases).

The limitations of our study include the relatively small number of cases investigated, given that it is a rare malignant tumor pathology and that this reflects work from a single tertiary center. Multicenter studies are needed to more thoroughly study the characteristics and prognostic factors associated with this tumor type and to generalize our results.

## 5. Future Directions

The factors that were associated with an unfavorable outcome were as follows: perivascular invasion, perineural invasion, lymph node invasion, skin invasion, and dimensions of the tumor more than 11 cm in diameter. The challenge in these giant CXPA is represented by skin invasion and large mass of the tumor. The skin invasion is changing the staging of the therapeutic approach of these giant tumors towards salvage surgery concepts. Moreover, the ulceration of the skin and a giant tumor mass require performing the surgery in complex multidisciplinary teams that can be available in tertiary units due to increased surgical time, risk of bleeding, and necessity to keep the patient for a longer period in the intensive care unit. The reconstruction is challenging due to the need to widen the skin defect due to the microscopic lymphatic invasion of the skin.

One answer to these problems could be the use of adjuvant immunotherapy before the surgical step. The idea of such approach could be derived from cases of melanomas involving the parotid region because melanoma immunotherapy is entering the stage of wide scale use [23]. Salivary duct carcinoma showed a robust response to nivolumab checkpoint inhibitor even in the presence of metastatic disease [24]. In 2023, a case of CXPA harboring HER-2 amplification had a survival of more than 3 years undergoing a regimen of targeted therapy combined with immunotherapy [25], In the future, such cases of giant CXPA could be submitted to personalized medicine trials centered on immunotherapy prior, during or after salvage surgery procedures.

## 6. Conclusions

Giant tumors of CXPA of the parotid gland are rare, derived from a primary or recurrent pleiomorphic adenoma of the parotid gland. The treatment of choice consists of ablative surgery and adjuvant radiotherapy. The therapeutic plan must be individualized and carried out by a multidisciplinary team composed of an imaging specialist, ENT surgeon, anesthetist, pathologist, radiotherapist, and oncologist. The factors that were associated with unfavorable evolution included the following: a tumor diameter over 11 cm, skin invasion, perivascular invasion, perineural invasion, and lymph node invasion. Prognosis is reserved, but a correct diagnosis and aggressive surgical management can increase survival rates.

## Data Availability

All data are available from the corresponding author upon reasonable request due to the increased size of the dataset.
